# 2138 Susceptibility to social influence is associated with alcohol self-administration and subjective alcohol effects

**DOI:** 10.1017/cts.2018.183

**Published:** 2018-11-21

**Authors:** Alyssa Schneider, Bethany Stangl, Elgin R. Yalin, Jodi M. Gilman, Vijay Ramchandani

**Affiliations:** National Institutes of Health

## Abstract

OBJECTIVES/SPECIFIC AIMS: Peer groups are one of the strongest determinants of alcohol use and misuse. Furthermore, social influence plays a significant role in alcohol use across the lifespan. One of the factors that most consistently predicts successful treatment outcomes for alcohol use disorders is one’s ability to change their social network. However, the concept of social influence as defined by suggestibility or susceptibility to social influence has not yet been studied as it relates to drinking behavior and acute subjective response to alcohol. Our objective was to examine the relationship between suggestibility and alcohol consumption and responses, using an intravenous alcohol self-administration (IV-ASA) paradigm in social drinkers. METHODS/STUDY POPULATION: Healthy, social drinkers (n=20) completed a human laboratory session in which they underwent the IV-ASA paradigm. This consisted of an initial 25-minute priming phase, where participants were prompted to push a button to receive individually standardized IV alcohol infusions, followed by a 125-minute phase during which they could push the button for additional infusions. IV-ASA measures included the peak and average breath alcohol concentration (BrAC) and number of button presses. Subjective responses were assessed using the Drug Effects Questionnaire (DEQ) and Alcohol Urge Questionnaire (AUQ) collected serially during the session. Participants completed the Multidimensional Iowa Suggestibility Scale (MISS) to assess suggestibility. The Alcohol Effects Questionnaire (AEFQ) was used to assess alcohol expectancies and the Timeline Followback questionnaire measured recent drinking history. RESULTS/ANTICIPATED RESULTS: After controlling for drinking history, greater suggestibility significantly predicted greater average BrAC, greater peak BrAC, and a greater number of button presses (*p*=0.03, *p*=0.02, *p*=0.04, respectively) during the early open bar phase. Suggestibility significantly predicted subjective alcohol effects following the priming phase which included “Feel,” “Want,” “High,” and “Intoxicated” and was trending for “Like” (*p*=0.02, *p*=0.03, *p*=0.01, *p*=0.03, *p*=0.054, respectively) as well as AUQ (*p*=0.03). After controlling for drinking history, suggestibility significantly predicted “Feel,” “Like,” “High,” and “Intoxicated” peak scores during the open bar phase (*p*=0.03, *p*=0.009, *p*=0.03, *p*=0.03, respectively). There was no association between suggestibility and “Want More” alcohol. Suggestibility was positively associated with three positive expectancies (global positive; *p*=0.04, social expressiveness; *p*=0.005, relaxation; *p*=0.03), and one negative expectancy (cognitive and physical impairment; *p*=0.02). DISCUSSION/SIGNIFICANCE OF IMPACT: These results indicate that social drinkers that were more suggestible had higher alcohol consumption, greater acute subjective response to alcohol, and more positive alcohol expectancies. As such, susceptibility to social influence may be an important determinant of alcohol consumption, and may provide insight into harmful drinking behavior such as binge drinking. Future analyses should examine the impact of suggestibility on alcohol-related phenotypes across the spectrum of drinking from social to binge and heavy drinking patterns.

